# Adjuvants Differentially Modulate the Immunogenicity of Lassa Virus Glycoprotein Subunits in Mice

**DOI:** 10.3389/fitd.2022.847598

**Published:** 2022-03-10

**Authors:** Albert To, Chih-Yun Lai, Teri Ann S. Wong, Madhuri Namekar, Michael M. Lieberman, Axel T. Lehrer

**Affiliations:** 1 Department of Tropical Medicine, Medical Microbiology, and Pharmacology, John A. Burns School of Medicine, The University of Hawai’i at Mānoa, Honolulu, HI, United States; 2 Pacific Center for Emerging Infectious Disease Research, John A. Burns School of Medicine, The University of Hawai’i at Mānoa, Honolulu, HI, United States

**Keywords:** Lassa virus (LASV), adjuvant, vaccine, antibody, cell mediated response, glycoprotein (GP)

## Abstract

Lassa Fever (LF) is an acute viral hemorrhagic fever caused by Lassa virus (LASV) that is primarily transmitted through contact with wild rodents in West Africa. Although several advanced vaccine candidates are progressing through clinical trials, some effective vaccines are virally vectored and thus require a stringent cold-chain, making distribution to rural and resource-poor areas difficult. Recombinant subunit vaccines are advantageous in this aspect as they can be thermostabilized and deployed with minimal storage and transportation requirements. However, antigen dose and adjuvant formulation must be carefully selected to ensure both the appropriate humoral and cell-mediated immune responses are elicited. In this study, we examine the immunogenicity of a two-step immunoaffinity-purified recombinant LASV glycoprotein (GP) with five clinical- and preclinical-grade adjuvants. Swiss Webster mice immunized intramuscularly with 2 or 3 doses of each vaccine formulation showed complete seroconversion and maximal GP-specific antibody response after two immunizations. Formulations with GPI-0100, LiteVax, Montanide™ ISA 51, and Montanide™ ISA 720 induced both IgG1 and IgG2 antibodies suggesting a balanced Th1/Th2 response, whereas formulation of LASV GP with Alhydrogel elicited a IgG1-dominant response. Splenocytes secreting both Th1 and Th2 cytokines i.e., IFN-γ, TNF-α, IL-2, IL-4 and IL-5, were observed from mice receiving both antigen doses formulated with ISA 720, LiteVax and GPI-0100. However, robust, multifunctional T-cells were only detected in mice receiving a higher dose of LASV GP formulated with GPI-0100. Our results emphasize the importance of careful adjuvant selection and lay the immunological basis for a recombinant subunit protein LF vaccine formulation.

## INTRODUCTION

Lassa Virus (LASV) is a highly pathogenic species of Old-World Arenavirus endemic to West Africa causing Lassa fever (LF), a potentially acute hemorrhagic disease, in infected individuals. LF is a zoonotic disease transmitted by direct or indirect contact with rodent hosts. It results in approximately 300,000–500,000 infections and nearly 5,000 deaths annually in West Africa, primarily documented throughout Nigeria and the Mano River Union (MRU) countries of Guinea, Sierra Leone, and Liberia (1–3). Secondary nosocomial transmission is also a serious route of infection in resource-deficient regions. Around 80-90% of infections are asymptomatic or mild, however, the mortality rate in hospitalized patients can reach 15% to 20% and upwards of 50% during epidemics (4). The uptick of LF in 16 Nigerian states during early 2021 constitutes the largest outbreak to date and recapitulates the need for a rapid response. Currently of the 6252 suspected LF cases, 1138 have been laboratory confirmed with 235 deaths, a case fatality rate (CFR) of 20.7% (5). Neurological sequelae, including encephalopathy and aseptic meningitis, and sensorineural hearing loss is common in recovered patients (6, 7).

LASV is morphologically pleotropic and its genome consists of 4 genes separated into two genomic segments responsible for viral structure, replication, and host antiviral evasion. There are no approved vaccines for LF and while there are several candidates in development, the diversity of vaccine platforms lags compared to other neglected tropical diseases, primarily consisting of recombinant or chimeric viral vectors (3, 8–15) as well as a DNA vaccine (16, 17). Furthermore, deployment of most virally vectored vaccines in endemic regions will be a logistical challenge as maintaining a proper cold chain can be difficult (18). Protein subunits are a reliable vaccine platform and can be administered either as a homologous prime-boost regimen or as boosters for other vaccines when immunity to the viral vector used is a concern. Stably transformed *Drosophila* S2 cell lines have been used to synthesize native-like viral antigens of several protein vaccine candidates against flavi- and filoviruses (19–24), despite having different glycosylation pathways than mammalian cells. The LASV glycoprotein complex (GPC) is the single viral antigen on the virion surface and is arranged as a trimeric spike of GP1-GP2 heterodimer units. It is a crucial target of the humoral and cellular immune response, recognized by antibodies and T-cells from LF survivors and seropositive individuals (25–27) and the lead antigen of all vaccine candidates (3, 8–12, 14, 15, 28). Formulation of the antigen with an appropriate adjuvant is critical in amplifying immunogenicity and modulating the type of response generated. Water-in-oil (W/O) emulsions are known to enhance the immunogenicity of protein antigens by depot formation, slowing the release of antigen while also protecting it against rapid degradation. Additionally, emulsions facilitate uptake by antigen presenting cells and incite inflammation at the injection site (29). Montanide™ ISA 51 (50% (v/v)) is based on a blend of mannide monooleate surfactant and mineral oil, whereas Montanide™ ISA 720 (70% (v/v)) uses a biodegradable non-mineral oil (30). Together they have been used in clinical trials involving cancer, AIDS, malaria, influenza and autoimmune vaccines and are very well tolerated (31). Additionally, these W/O emulsions have been shown to induce IgG isotype switching and IFN-γ secretion in mice and non-human primates (NHP) (30, 32, 33). The mechanism of action includes the slow release of antigen and diffusion of oil droplets to draining lymph nodes (31). LiteVax is a preclinical adjuvant consisting of carbohydrate fatty acid sulphate esters (CFASE), chemically resembling monophosphoryl lipid A but modified to reduce reactogenicity and is formulated in a squalane-in-water emulsion (34). Immunomodulation has been attributed to the upregulation of costimulatory molecules and the production of proinflammatory cytokines by dendritic cells (35). CFASE have been used previously for veterinary purposes (36) and in phase IIa clinical trials in formulations of two peptide-protein vaccines with a dose of up to 10 mg (37–39). By contrast, GPI-0100 is a stable semisynthetic triterpenoid saponin derived from *Quillaja saponaria* extract, which has been used in clinical trials of cancer and viral vaccines with dosing up to 3 mg (40–43). Similarly, saponins stimulate pro-inflammatory molecules which recruit dendritic cells, promote antigen processing in the cytosol and NLRP3 inflammasome activation (44, 45). Both are known to promote Th1 immunity, stimulating antigen specific-cytotoxic T-cells and antibody production against poorly immunogenic antigens (34, 42, 46). A formulation inducing a multifunctional T-cell response and a diversified antibody response against the LASV GP can be considered a promising LF vaccine candidate for further development. In this study we examine both the humoral and cellular responses to a S2 cell derived, highly purified soluble LASV GP coadministered with these clinical and pre-clinical grade adjuvants, in comparison to Alhydrogel (alum), in Swiss Webster mice.

## MATERIALS AND METHODS

### Ethical Statement

All mouse experiments were approved by the University of Hawaii Institutional Animal Care and Use Committee (IACUC), and conducted in strict accordance with local, state, federal, and institutional policies established by the National Institutes of Health and the University of Hawai’i IACUC. The University of Hawai’i, John A. Burns School of Medicine (JABSOM) Laboratory Animal Facility is accredited by the American Association for Accreditation of Laboratory Animal Care (AAALAC). All animal experiments were conducted in consultation with veterinary and animal care staff at the University of Hawai’i. Mice were bred in colonies at JABSOM from original stocks obtained from Taconic Bioscience (Hudson, NY).

### Expression and Purification of LASV GP Antigen

The transmembrane domain-deleted, native sequence gene cassette of the Josiah Strain LASV GP (Genbank accession number NP_694870.1) was generated using conventional PCR amplification of an expression vector obtained from the United States Army Medical Research Institute of Infectious Diseases (USAMRIID) to encode amino acids 59-427. The gene cassette was inserted using restriction enzyme cloning downstream of the BiP signal on the pMT-Bip expression vector (Invitrogen, Carlsbad, CA) for secretion into the cell culture supernatant. ExCell420 medium (Sigma-Aldrich, St. Louis, MO) -adapted *Drosophila* S2 cells were cotransfected with the expression vector and selectable marker plasmid pCoHygro using Lipofectamine LTX with PLUS reagent (Invitrogen, Carlsbad, CA) following manufacturer’s instructions. Stably transformed cell lines were created with ExCell420 containing hygromycin B (Invivogen, San Diego, CA) at 200 μg/mL for 4 weeks in 100 mm tissue culture-treated cell culture dishes (Corning, Corning, NY). Transformed cell lines were induced with culture medium containing 200 μM CuSO_4_ for one week. This concentration has been optimized based on the growth kinetics of similarly generated cell lines (21, 24, 47). The presence of GP in the supernatant was verified by sodium dodecyl sulphate polyacrylamide gel electrophoresis (SDS-PAGE) and western blotting.

The antigen was produced in a WAVE Bioreactor (GE Healthcare, Piscataway, NJ) using a 2L bag (1L culture volume) and was subsequently purified by immunoaffinity chromatography (IAC) using the anti-GP1 monoclonal antibody (mAb) R3621 (generated and provided by the USAMRIID). Purified antibody was coupled to a 1- or 5-mL HiTrap NHS-activated HP column (GE Healthcare, Piscataway, NJ) at 2 or 10 mg/mL, respectively, according to the manufacturer’s protocol for use in antigen purification. The S2 cell culture medium containing recombinant protein was clarified by centrifugation at 3210 x g for at least 15 min and filtered (0.2 μm pore size) before being loaded onto the IAC column at a linear flow rate of approximately 1 or 3 mL/min with the 1- or 5-mL columns, respectively. The resin was washed with PBS containing 0.05% (v/v) Tween-20 (PBST, 140 mM NaCl) followed by PBS at a flow rate of 1 or 5 mL/min, respectively. Bound protein was eluted from the IAC column with 20 mM glycine buffer, pH 2.5, at the same flow rate into a fraction collector. The eluate was neutralized with 1M phosphate buffer, pH 7.4, buffer exchanged into PBS and concentrated using Amicon® Ultra-15 devices with a 30 kDa cutoff (EMD Millipore, Billerica, MA).

The concentrated and buffer-exchanged eluates from the IAC column were loaded onto a HiLoad 16/600 column (GE Healthcare, Piscataway, NJ) equilibrated with PBS and separated at a flow rate of 1.0 mL/min. The antigen collected from fractions containing oligomeric and GPC protomer were pooled and separated from fractions containing monomeric GP1 and GP2 subunits by size exclusion chromatography (SEC). The purified product was analyzed by SDS-PAGE with Coomassie blue staining in parallel to western blotting and quantified by UV absorption. Purified recombinant proteins in PBS were filter sterilized (0.2 μm pore size) and stored at −80°C.

### Coomassie Stained Polyacrylamide Gel Electrophoresis and Western Blot

Purified GP fractions were mixed with NuPAGE LDS sample buffer (Thermofisher, Waltham, MA), boiled for 10 minutes and separated on NuPAGE™ 4-12% Bis-Tris Protein Gels (Thermofisher, Waltham, MA). Subsequently, gels were washed in deionized water three times for 5 minutes, stained with SimplyBlue™ SafeStain (Thermofisher, Waltham, MA) for one hour and destained overnight with distilled water. For western blotting, resolved protein from an unstained gel was transferred to nitrocellulose membranes by Bolt Mini Blot Module (Thermofisher, Waltham, MA). The membranes were blocked with TBS containing 4% non-fat milk powder for 1 hour at RT and subsequently probed with a panel of characterized anti-LASV GP human monoclonal antibodies (48) (provided by the Walter Reed Army Institute of Research) at a 1:1,000 dilution in Tris-buffered saline with 0.2% Tween-20 (v/v) (TBST) (pH 7.2) containing 4% non-fat milk powder overnight at 4°C with gentle agitation. After three 5-minute washes with TBST, the membranes were incubated with IRDye® 800CW goat anti-human IgG secondary antibody (Li-Cor, Lincoln, NE) at a 1:10,000 dilution for another hour. The membranes were washed three times with TBST and imaged using an Odyssey® CLx Imaging System and processed using Image Studio™ (Li-Cor, Lincoln, NE).

### Immunization, Serum Collection and Splenocyte Harvest

Swiss Webster (SW) mice (age 6-10 weeks old) were immunized intramuscularly (i.m.) in the hind legs with 3 doses of 10 μg LASV GP on Days 0, 21 and 42, or 2 doses of 2 or 10 μg LASV GP on Days 0 and 14 in the absence or presence of the adjuvants as follows: Alhydrogel® adjuvant 2%, “alum” (100 μg per dose) (*In vivo*Gen, San Diego, CA), GPI-0100 (250 μg per dose) (Hawaii Biotech Inc, Honolulu, HI), Montanide™ ISA 51 (50%(v/v)) (Seppic, Fairfield, NJ), Montanide™ ISA 720 (70%(v/v)) (Seppic, Fairfield,NJ), or LiteVax (1mg per dose) (LiteVax BV, Ophemert, the Netherlands). Each vaccine formulation and recommended dose per mouse were prepared according to the adjuvant manufacturer’s instructions and based on experience from earlier vaccine immunogenicity studies (21, 31, 34). Sterile PBS was used to dilute the LASV GP when needed. Adjuvant alone formulations were mixed with the same sterile PBS instead of antigen solution. Sera were collected two weeks after each immunization by tail bleeds or cardiac puncture for terminal bleeds, and mouse spleens were harvested one week after the final immunization. Splenocyte suspensions were prepared by mechanical dissociation using a gentleMACS™ Dissociator (Miltenyi Biotec, Auburn, CA). Red blood cells were lysed using eBioScience™ RBC lysis buffer (Thermo Fisher Scientific, Waltham, MA), and the splenocytes were cryopreserved in fetal bovine serum (FBS) + 10% Dimethyl sulfoxide (DMSO).

### Coupling of Microspheres With Recombinant LASV GP Antigens

The coupling of microspheres with glycoprotein antigen was performed as described previously (49). Internally dyed, carboxylated, magnetic microspheres (MagPlex-C ™) were obtained from Luminex Corporation (Austin, TX, USA). A two-step carbodiimide process recommended by Luminex was used to covalently couple 25 μg of purified antigen to the surface of 5×10^6^ microspheres. The antigen-conjugated microspheres were stored in 800 μL of PBN buffer (PBS with 1% bovine serum albumin Fraction V, OmniPur, and 0.05% sodium azide (Sigma-Aldrich, St. Louis, MO)) at 4°C. Microspheres dyed with spectrally different fluorophores were also coupled with bovine serum albumin (BSA) to serve as a control for nonspecific protein binding.

### Microsphere Immunoassay

The microsphere immunoassay was performed as previously described with modifications (47, 50). Microspheres coupled with recombinant LASV GP and BSA were pooled in PBS-1% BSA, 0.02% Tween-20 (PBS-BT) at a dilution of 1:200. Fifty microliters of the antigen-coupled microsphere suspension (1:200) were added to each well of black-sided 96-well plates. Serum samples were diluted 1:8,000 in PBS-BT for total IgG or 1:1,000 for determination of IgG subclasses, and added to the microspheres (1:1) in duplicate before incubation for 3 hours on a plate shaker set at 700 rpm in the dark at 37°C. The plates were then washed two times with PBS-BT using a magnetic plate separator (Millipore Corp., Billerica, MA). Red-phycoerythrin (R-PE) conjugated F(ab’)2 fragment goat anti-mouse IgG (Jackson Immunoresearch, West Grove, PA), or anti-mouse IgG1, IgG2a, IgG2b or IgG3 conjugated Human adsorbed-PE (Southern Biotech, Birmingham, AL) were added at 1 μg/mL (50 μL) to the plates and incubated for another hour. The plates were washed two times, and microspheres were resuspended in 120 μL of drive or sheath fluid and analyzed on a Magpix or Luminex instrument, respectively (Luminex Corporation, Austin, TX). Data acquisition detecting the median fluorescence intensity (MFI) was set to a minimum acquisition from 50 beads per spectral region. Antigen-coupled beads were recognized and quantified based on their spectral signature and signal intensity, respectively. Cutoff values were calculated as the average MFI of 5 serum samples from five naive SW mice plus three standard deviations. Serum samples with MFI values greater than the cutoff were considered positive. The signals generated by the control BSA-coupled beads were all below the assay cutoffs.

### Recombinant Vesicular Stomatitis Virus-LASV GP (rVSV-LASV GP) Neutralization Assay

Replication-competent rVSV expressing LASV GP (Josiah strain) was obtained from the University of Texas Medical Branch (UTMB) (3) and the virus stocks were amplified in Vero cells. For the plaque reduction neutralization test (PRNT), pooled mouse serum samples were heat-inactivated at 56°C for 30 minutes. 19.7E, a known neutralizing mAb, was used as a positive neutralization control, starting at a concentration of 20 μg/mL. Six 3-fold serial dilutions of serum samples (and mAb), starting at a 1:10 dilution, were prepared and incubated with 50 plaque-forming units (PFU) of rVSV-LASV GP at 37°C for 1 hour. Antibody-virus complexes were added to Vero cell monolayers in 6-well plates and incubated at 37°C for another hour. Following addition of DMEM media containing 2% FBS and 1% agarose and incubation for 72 hours, cells were fixed and stained with a solution containing 1% formaldehyde, 1% methanol, and 0.05% crystal violet for 2 hours for plaque enumeration. The neutralization titers (PRNT_50_) were defined as the serum dilution or mAb concentration that resulted in 50% infectivity reduction using a constrained sigmoidal 4PL nonlinear regression model (GraphPad Software version 9, San Diego, CA).

### FluoroSpot Assay

The FluoroSpot assay was performed using mouse IFN-γ/TNF-α/IL-2 Triple-Color FluoroSpot kits (Cellular Technology Limited (CTL), Cleveland, OH). Splenocytes were rested in a 37°C, 5% CO2, humidified incubator for 3 hours after thawing in RPMI-1640 medium supplemented with 10% FBS, penicillin (100 units/mL) and streptomycin (100 μg/mL) (RPMI-10). A total of 3.0 x 10^5^ splenocytes per well in serum-free CTL-Test™ medium (Cellular Technology Limited, Shaker Heights, OH) were added in a 96 well PVDF membrane plate pre-coated with capture monoclonal antibodies and stimulated for 24 hours at 37°C, 5% CO2 with pools of 15-mer peptides with 11 overlapping amino acids (5 μg/mL per peptide), covering the entire LASV GP (GenScript, Piscataway, NJ). A cell activation cocktail (BioLegend, San Diego, CA) containing 40.5 μM phorbol myristate acetate (PMA) and 669.3 μM ionomycin was used at 1:500 dilution as the positive control and culture medium as the negative control. Each stimulation condition was set up in duplicates, and the costimulatory anti-CD28 antibody was added to the cells prior to incubation at a final concentration of 0.1μg/mL. Plates were developed using specific monoclonal detection antibodies (anti-murine IFN-γ (Biotin), anti-murine TNF-α (Hapten1) and anti-murine IL-2 (FITC)) and fluorophore-conjugated secondary reagent (Strep CTL-Red™, anti-FITC Alexa Fluor® 488 and anti-hapten1 CTL-Yellow™)). The spots were enumerated using the CTL ImmunoSpot® S6 Universal Analyzer (Cellular Technology Limited, CTL, Cleveland, OH) and analyzed by the CTL ImmunoSpot® 7 Software. The number of antigen specific cytokine-secreting spot forming cells (SFCs) per million cells was calculated by subtracting the number of spots detected in the medium only wells. The polyfunctional T-cell response was determined as the percentages of cells secreting two or three cytokines.

### Cytokine Secretion Multiplex Assay

Concentrations of IFN-γ, TNF-α, IL-2, IL-4, and IL-5 cytokines in supernatants from splenocytes re-stimulated with whole LASV GP antigen were measured using a quantitative MILLIPLEX® MAP Mouse Cytokine/Chemokine Magnetic Bead Panel Multiplex Assay (Millipore Sigma, Burlington, MA) according to the manufacturer’s instructions. Splenocytes were rested in a 37°C, 5% CO2, humidified incubator overnight after thawing in RPMI-10. A total of 3.0 x 10^5^ cells per well in 200 μL in RPMI-10 were plated into a 96-well, round-bottomed culture plate, and incubated with 10 μg/mL of LASV GP or medium. Potential endotoxins in the antigen preparation were removed using a Pierce™ High-Capacity Endotoxin Removal Spin Column (Thermo Fisher Scientific, Waltham, MA) prior to stimulation. Each stimulation condition was set up in duplicate, and cell culture supernatants were harvested after 48 and 96 hours. The supernatants from stimulation duplicates were pooled and tested undiluted for cytokines. MFI was measured using the Luminex 200 xMAP system (Millipore) and data were analyzed using the Luminex xPONENT software (Millipore). Standards were prepared using a series of five-fold dilutions as suggested by the manufacturer. The concentration of each cytokine in the culture supernatants was calculated using a logistic 5-parameter curve-fitting formula in the software based on the curve generated by the standards provided by the manufacturer.

### Statistical Analysis

Determination of significant differences in IgG antibody titers between primary and subsequent immunizations of the mice from the same group was done using a two-way ANOVA followed by a Tukey’s multiple comparison. Significant differences in total antigen-specific IgG and IgG subclasses between groups were determined using a two-way ANOVA followed by Tukey’s multiple comparison test to compare titers of the same antigen dose across adjuvants, and antigen dosing between formulations using the same adjuvant. Significant differences in cytokine secretion were determined using a two-way ANOVA followed by Tukey’s multiple comparison test to compare the number of antigen-specific splenocytes and differences in cytokine concentrations in the stimulated culture supernatants between vaccine formulation groups. P< 0.05 was considered significant in all instances. All statistical analyses were performed with a commercial statistical program (Prism, Graphpad Software, San Diego, CA).

## RESULTS

### Potently Neutralizing Epitopes Are Preserved on a Soluble LASV GP

**FIGURE 1 |:**
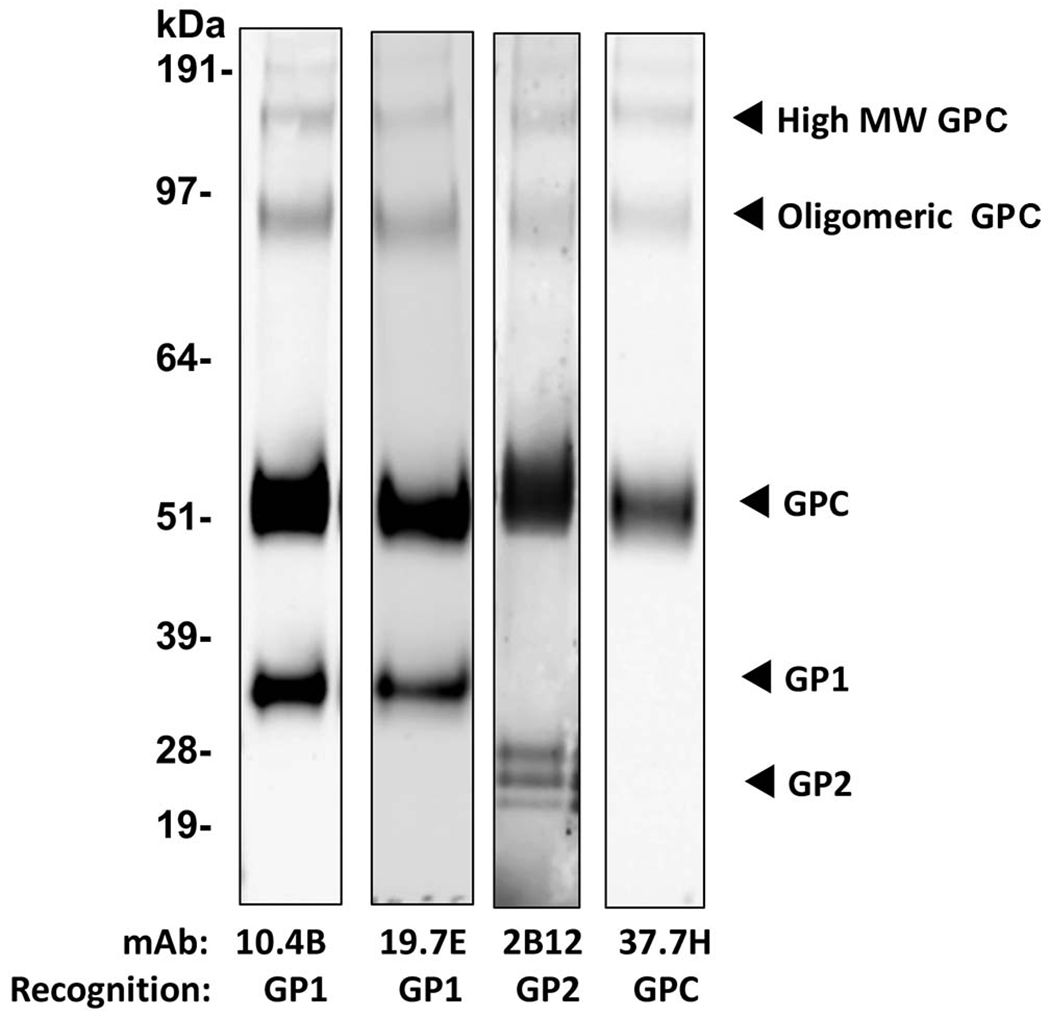
Recombinant LASV GP protein expressed in Drosophila S2 cells after purification using IAC with R3621 mAb followed by SEC. Western blot of purified LASV GP. Four μg of the vaccine antigen was probed with mAb 10.4B, 19.7E, 2B12, and 37.7H targeting epitopes on GP1, GP2, and GPC, at a dilution of 1:1000.

Drosophila S2 cell-produced LASV GP purified by immunoaffinity chromatography (IAC) using a GP1-specific monoclonal antibody (mAb), followed by a secondary size-exclusion step to remove smaller subunits (Supplementary Figure 1A), appeared as intact GP-complex (GPC) or higher oligomers on Coomassie-stained SDS-PAGE, however, on a western blot, additional lower molecular bands representing GP1 and GP2 subunits also appeared. The denaturing conditions of SDS-PAGE and sensitivity of the monoclonal antibodies utilized likely contributed to the appearance of monomeric GP1 and GP2 despite being only minor components (estimated as less than 10%) of the final purified antigen (fractions 5 and 6 in Supplementary Figure 1B). The lower molecular weight bands correspond to the GPC protomer adopting the post-fusion conformation where GP1 is shed from the GP2 trimer core, as the association between GP1 and GP2 is non-covalent (51) and is thus susceptible to physical and chemical disruption. Utilizing well-characterized human mAbs derived from LASV-infected patients (48) confirmed key epitopes, including some neutralizing epitopes, on GP1, GP2 and GPC are preserved on the recombinant antigen, suggesting that IAC purification with a low pH elution does not adversely impact the conformation of the GP (Figure 1). The GP1 subunit on the western blot, recognized by conformational mAb 10.4B and 19.7E, is present both as a monomer and associated with GP2, while the GP2 subunit, recognized by 2B12, appears as multiple, lower molecular weight species likely due to differential glycosylation during synthesis (52). Furthermore, the single band appearing at ~51kDa and higher molecular weight species consisting of both GP1 and GP2 subunits that are both reactive to the mAb 37.7H suggest that the integrity of a crucial, pre-fusion neutralizing epitope is also preserved in this antigen preparation.

### Soluble LASV GP Is Immunogenic in Mice

**FIGURE 2 |:**
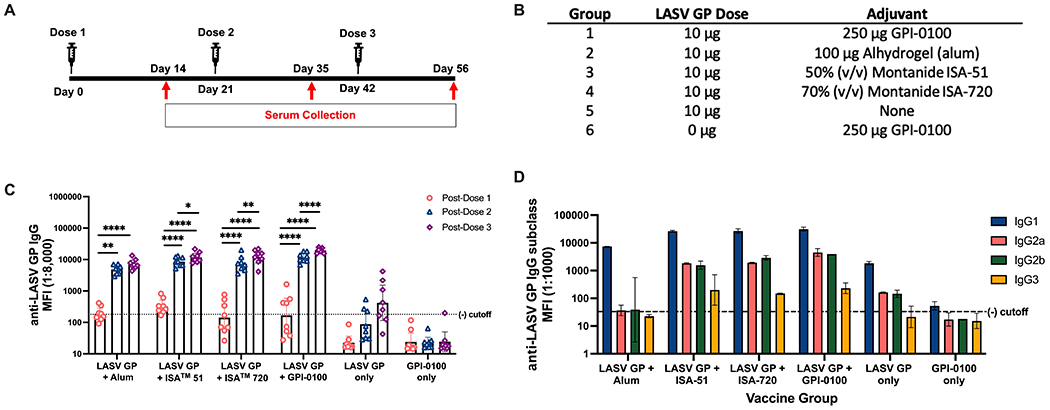
Total IgG and IgG subclasses generated in a 3-dose study of adjuvanted LASV GP in SW mice. **(A)** Vaccination schedule. **(B)** Experimental groups. **(C)** Geometric mean titers (GMT) with 95% CI of LASV GP-specific IgG MFI of serum samples from all groups of mice immunized with each formulation (n=8 for each group). Each symbol represents the mean of duplicates from individual mice. A two-way ANOVA followed by a Tukey’s multiple comparison was used to determine statistical differences in IgG titers between primary and subsequent doses. **(D)** GMT with 95% CI of LASV-GP specific IgG subclass MFI of pooled sera from experimental groups post-dose 3. Statistical significance was not calculated as each data point consisted of a single pooled sample ran in duplicates (*p < 0.05, **p < 0.01, ****p < 0.0001).

Given that presumptive protective epitopes are preserved on S2 cell derived LASV GP, 10 μg of antigen were formulated with four adjuvants: 1) Alhydrogel (alum), 2) Montanide™ ISA 51, 3) Montanide™ ISA 720 and 4) GPI-0100 (Figures 2A, B). LiteVax was acquired later in this study and was not tested in the first immunogenicity experiment. SW mice were immunized with 3 doses of each vaccine formula and sera were collect two weeks after each dose (Figure 2C). Antibody titers were measured using an antigen-coupled microsphere immunoassay and expressed as the GMT +/− 95% CI of MFI. Marked seroconversion for most groups can be seen two weeks after the second immunization, and a third dose did modestly boost IgG titers for all groups except the group receiving alum. The group receiving the LASV GP + GPI-0100 demonstrated the largest boost after the third dose where the titers increased by approximately 2-fold. Surprisingly, 3 doses of the LASV GP alone did elicit seroconversion in >50% of the mice at a level greater than the titers seen after a single adjuvanted dose, but titers were still approximately two orders of magnitude lower than the titers produced after a second adjuvanted dose. No or low levels of neutralizing antibodies (titer <10) were detected using a surrogate PRNT with a recombinant vesicular stomatitis virus (rVSV) expressing the LASV GP of the Josiah strain (rVSV-LASV GP) (Supplementary Figure 2A, B).

As the effector function of non-neutralizing antibodies has been implicated with vaccine-induced protection (53), establishing the degree of IgG subclass diversity induced by each adjuvant might be beneficial for selecting a final vaccine formulation for further testing. IgG subclass diversity was determined from pooled post-dose 3 sera and revealed a predominantly IgG1 response with IgG2a, IgG2b and IgG3 produced in groups given formulations with GPI-0100, ISA 51 and 720 (Figure 2D). The highest IgG2a and IgG2b MFIs were seen in the group given the GPI-0100 formulation, comprising about 11% and 10% of total IgG, respectively. ISA 51 and 720 engendered similar levels of IgG1 but differed in their ability to generate IgG2 subclasses. The IgG2a and IgG2b titers of the group immunized with the ISA 720 formulation are comparable to the group given GPI-0100 at 6% and 9% of the total IgG, respectively, whereas ISA 51 produced IgG2 subclasses at 6% and 5%, respectively. Mice given the formulation using alum produced exclusively IgG1 indicating that this adjuvant drives a Th2-type immunological response, a noteworthy switch since LASV GP alone did generate detectable IgG2 subclass titers. Low levels of IgG3 were observed across adjuvant groups. Based on total IgG titer generated and evidence of diverse IgG subclass induction, the formulations with ISA 720 and GPI-0100 were chosen for further study. LiteVax was added as another adjuvant option in the following experiments.

### Two Doses of an Adjuvanted LASV GP Generate a Robust IgG Response

**FIGURE 3 |:**
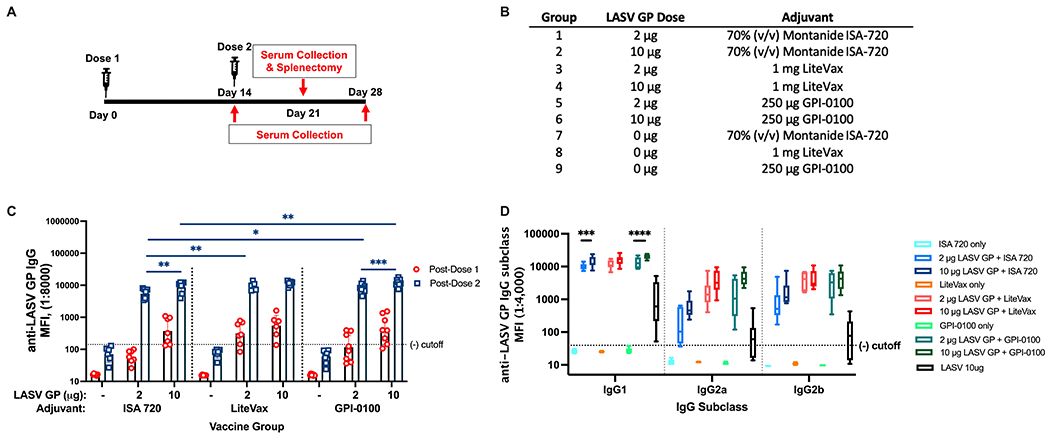
Total IgG and IgG subclass titers from a 2-dose study with 2 or 10 μg of LASV GP formulated with select adjuvants. **(A)** Vaccination schedule. **(B)** Experimental groups. **(C)** Geometric means (GMT) with 95% CI of LASV GP-specific IgG MFI of serum from all groups of mice immunized with each formulation. Mice were given either 2 or 10 μg of antigen formulated with adjuvants selected from Figure 2. Each symbol represents the mean of duplicates from individual mice (n=7 or 8 for each group). A two-way ANOVA followed by a Tukey’s multiple comparison was used to determine statistical differences across antigen dose and adjuvant formulations **(D)** The median of individual MFIs plotted with 5-95 percentile of LASV GP-specific IgG subclasses from each mouse group. Statistical differences between titers of the same IgG subclass across vaccine formulations was calculated using a two-way ANOVA followed by Tukey’s multiple comparison (*p < 0.05, **p < 0.01, ***p < 0.001, ****p < 0.0001).

To determine if selected adjuvants can have a dose sparing effect after two doses and alter the IgG2/IgG1 subclass ratio, groups of mice received either ISA 720, GPI-0100 or LiteVax formulated with either 2 or 10 μg of LASV GP (Figures 3A, B). After a single dose, 10 μg of GP with an adjuvant generated higher total IgG titers than respective formulations employing 2 μg of GP for all adjuvants (Figure 3C). Mice immunized with the 10 μg GP + LiteVax formulation showed the highest MFI at this time point. After the second dose, 10 μg GP + GPI-0100 induced statistically higher IgG titers than the group receiving 10 μg GP + ISA 720, while 2 μg of GP with LiteVax or GPI-0100 were more effective than 2 μg of GP + ISA 720. An antibody dose sparing effect was only noticed in groups receiving LiteVax (no difference in antibody titers between groups immunized with 2 or 10 μg of antigen), while 10 μg of GP with ISA 720 or GPI-0100 elicited statistically higher IgG titers than 2 μg. Overall, the second dose boosted total IgG titers of most antigen-immunized groups by 30- to 42-fold, and by 100- and 94-fold for the groups receiving 2 μg of GP with ISA 720 or GPI-0100, respectively.

**FIGURE 4 |:**
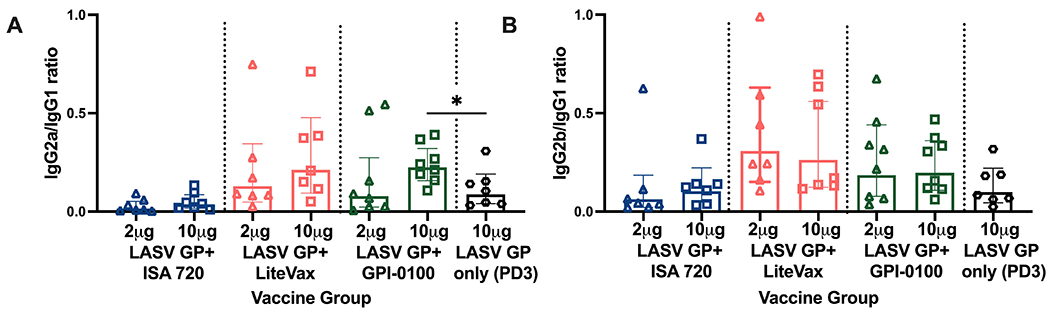
IgG subclass ratios of immunized mice from experimental groups. **(A)** IgG2a/IgG1 and **(B)** IgG2b/IgG1 ratio. Geometric means (GMT) with 95% CI of LASV GP-specific IgG subclass MFI ratio of serum from all groups of mice immunized with each formulation from Figure 3B. PD2 except for LASV GP alone (PD3). Each symbol represents the mean of duplicates from individual mice (n=7 or 8 for each group). A two-way ANOVA followed by a Tukey’s multiple comparison was used to determine statistical differences in subclass ratios compared to LASV GP alone (*p < 0.05).

IgG1 titers followed the same trend as total IgG. Two and 10 μg GP formulated with LiteVax showed comparable MFIs, while 10 μg of GP with ISA 720 or GPI-0100 yielded higher MFI values than 2 μg of GP (Figure 3D). The MFI values of IgG2a and IgG2b across antigen-immunized groups were not statistically different due to the high variation seen within the same group, although the highest IgG2a and IgG2b MFI GMTs were observed in mice immunized with 10 μg GP + LiteVax or GPI-0100, and the lowest IgG2 subclass MFIs were observed in the 2 μg GP + ISA 720 group. Again, it appears that the antigen dose did not significantly affect the generation of IgG2a and IgG2b titers if LiteVax was coadministered, suggesting a dose-sparing effect of this adjuvant. To determine if two doses of each test formulation differ in terms of subclass induction, the IgG2a/IgG1 and IgG2b/IgG1 ratios for each mouse were calculated (Figures 4A, B). No remarkable difference was observed between the immunized groups although the antigen formulations containing LiteVax or GPI-0100 showed higher ratios than the ISA 720 formulations. Compared to the IgG2a/IgG1 ratios from mice immunized with 3 doses of LASV GP alone, two doses of the ISA 720 formulations drove a humoral response favoring IgG1, a noticeable difference as 3 doses generated IgG2a (and IgG2b) levels at an order of magnitude higher. The only formulation to significantly alter the IgG2a/IgG1 ratio compared to GP alone (after 3 doses) was the formulation containing 10 μg GP + GPI-0100. No significant difference in the IgG2b/IgG1 ratios was observed compared to GP alone. Overall, adjuvants LiteVax and GPI-0100 produced a more balanced IgG subclass response independent of antigen dose.

### Two Doses of an Adjuvanted LASV GP Generate a Robust Cell Mediated Immune Response

**FIGURE 5 |:**
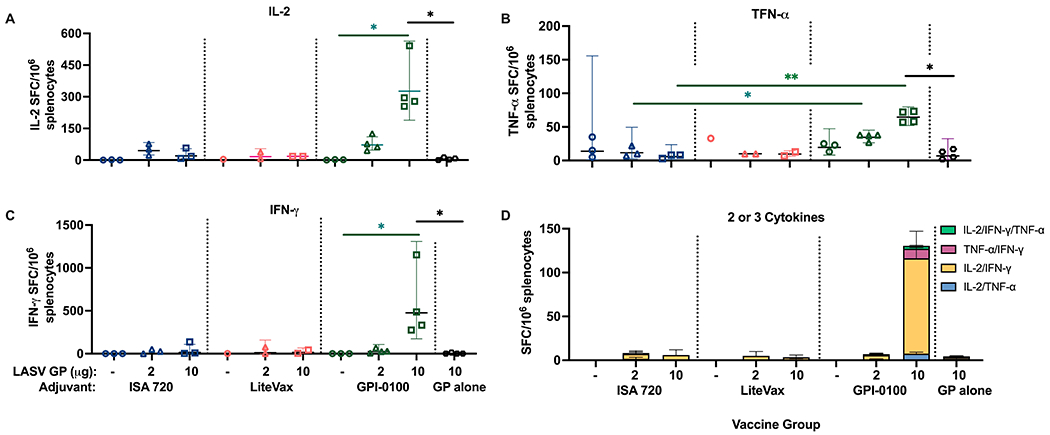
Cytokine secretion measured from mouse splenocytes using a FluoroSpot assay. Geometric mean (GMT) with 95% confidence intervals of **(A)** IL-2, **(B)** TNF-α, **(C)** IFN-γ and **(D)** polyfunctional cytokine secretion from splenocytes harvested from mouse groups one-week post-dose 2 and stimulated with a LASV GP peptide pool for 24 hours. (n=3 for the ISA 720 groups, n=1-2 for LiteVax groups, and n=3-4 for GPI-0100 groups) A two-way ANOVA, followed by a Tukey’s multiple comparison was used to compare the number of cytokine-secreting cells from immunized groups to the group receiving LASV GP alone, and between different adjuvant formulations of the same antigen concentration, respectively. (*p < 0.05, **p < 0.01).

As robust cell mediated immunity is considered a correlate of protection against LASV infection and may be associated with viral clearance (54, 55), it was important to determine if two doses of our vaccine formulations can induce GP-specific polyfunctional T-cells. IL-2, TNF-α, and IFN-γ secretion were measured using a multi-parametric FluoroSpot assay of splenocytes harvested from half the mice in each vaccine group taken one week after the second dose. Splenocytes were stimulated with a LASV GP peptide pool for 24 hours. Mice immunized with 10 μg GP + GPI-0100 were the only group to show a significantly higher number of single cytokine secreting cells than mice receiving adjuvant alone or 2 μg of GP with the same adjuvant (Figures 5A–D). Similarly, this group had significantly higher numbers of single cytokine secreting cells than other 10 μg GP formulations with different adjuvants. Unsurprisingly, the 10 μg GP + GPI-0100 group was the only group to also show a solid polyfunctional T-cell response of lymphocytes secreting 2 or 3 cytokines, suggesting that this formulation may elicit potent cellular immunity.

**FIGURE 6 |:**
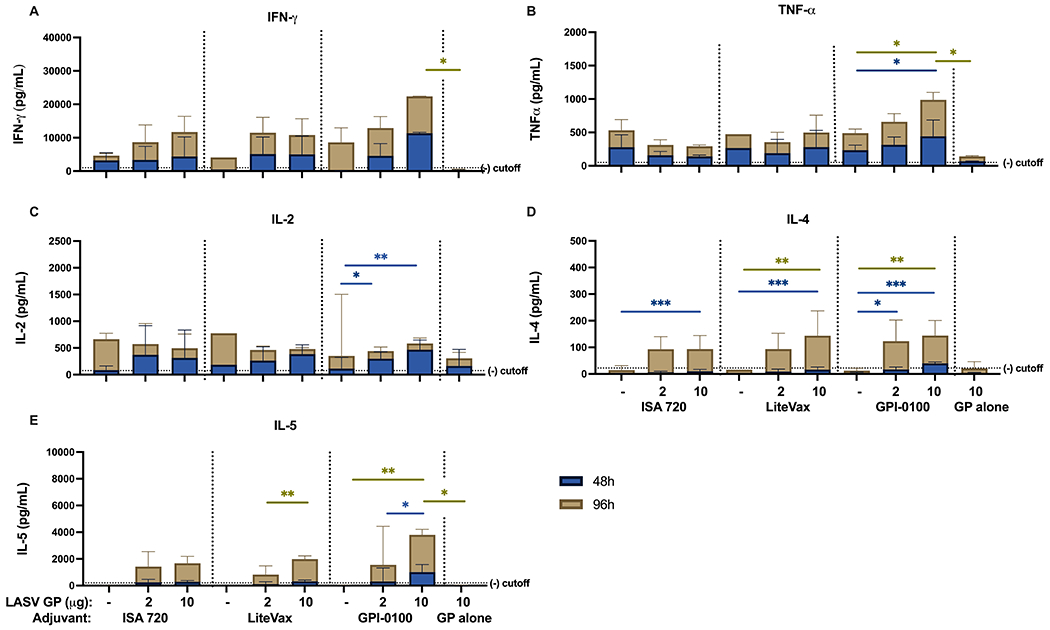
Cytokine secretion measured from mouse splenocytes using Multiplex Cytokine assay **(A)** IFN-γ, **(B)** TNF-α, **(C)** IL-2, **(D)** IL-4 and **(E)** IL-5 cytokine secretion from splenocytes harvested from mouse groups one-week post-dose 2 and stimulated with LASV GP antigen for 48 (blue) and 96 hours (gold). Bars indicate the GMT with 95% confidence intervals. (n=3 for the ISA 720 groups, n=2-3 for LiteVax groups, and n=3-4 for GPI-0100 groups) A two-way ANOVA, followed by a Tukey’s multiple comparison was used to compare the secreted cytokine concentrations from immunized groups compared to mice receiving LASV GP alone and between different adjuvant formulations of the same antigen concentration, respectively. (*p < 0.05, **p < 0.01, ***p < 0.001).

This finding was further supported using a multiplex cytokine secretion assay of splenocytes stimulated with LASV GP antigen. Cellular responses from all vaccine groups could be detected at 96h, if not earlier, although statistically significant differences in cytokine secretion could not be seen between groups receiving ISA 720 or LiteVax formulations, except in IL-4. Higher concentrations of IFN-γ and TNF-α, at 48h and 96h, respectively, were observed from splenocytes from mice immunized with the 10 μg GP + GPI-0100 formulation than from splenocytes of mice given a lower antigen dose and/or with ISA720 or LiteVax (Figures 6A–E). Th2 cytokines IL-4 and IL-5, which play a role in B- and T-cell activation, were also detected in animals receiving 10 μg GP + GPI-0100 at 48h and 96h post-stimulation, respectively, and were elevated compared to groups receiving the same antigen dose but with a different adjuvant. An IL-2 response was only detected in the groups given antigen (2 and 10 μg) and GPI-0100.

## DISCUSSION

An advantage of recombinant subunit protein vaccines compared to virally vectored vaccine platforms is that the lead antigen may be thermostabilized alone or together with an adjuvant to withstand a wider range of temperatures thus easing the logistics of distribution and deployment especially to resource-poor and isolated regions where maintaining a cold chain is difficult (56, 57). The non-infectious nature of this platform also does not exclude the medically vulnerable (e.g., immunocompromised populations), thereby increasing recipient reach (19, 58–60). Expression of antigens in stably transformed *Drosophila* S2 cell lines requires less stringent culturing conditions than mammalian cell lines while using immunoaffinity chromatography for purification can significantly improve purity, thereby reducing the number of required purification steps and reducing production costs (58, 59). Insect cell-derived viral surface glycoproteins formulated with adjuvant have a good safety and immunogenicity record in clinical trials (61–64).

We have shown that robust humoral and cellular immunity in outbred SW mice can be generated using insect-cell derived, recombinant LASV GP purified using IAC and SEC, when paired with the appropriate adjuvant. Clinical and pre-clinical grade adjuvants tested in this study generated a strong GP-specific antibody response as early as a single dose with some formulations. Near maximal titers induced upon the second dose were boosted only slightly by a third vaccine dose. Compared to alum, which skews the immune response towards a Th2 response, the adjuvants ISA 51, ISA 720, LiteVax and GPI-0100 produced a diversified antibody response which included different IgG subclasses, suggesting a balanced humoral response with greater potential for opsonization and complement fixation through Fcγ receptor interaction. Unlike IgG1, IgG2a and 2b can also activate FcγRIV, which is found on monocytes, macrophages and neutrophils, and is the predominant receptor involved in antibody-dependent cellular cytotoxicity (ADCC) or phagocytosis (ADCP) (65). LASV GP-specific, but non-neutralizing IgG antibodies have been implicated as a survival correlate in both rabies- (LASSARAB) and measles-vectored (MeV-NP) LF vaccines (53, 66).

Although our LASV GP antigen displays known neutralizing epitopes, a surrogate PRNT using rVSV-LASV GP on pooled sera from mice given three doses of vaccine did not show detectable levels of neutralizing antibodies. The role of polyclonal neutralizing antibodies in vaccine efficacy is not well understood as they do not appear to be correlated with survival in primary LF (1) nor specifically required for vaccine efficacy (53, 66). Acute LF patients usually develop only low levels of LASV-specific antibodies, while a subset of convalescent patients have been reported with a mature antibody repertoire, including potent virus neutralizing titers, that is highly divergent from the germline (67, 68). It is therefore conceivable that vaccine recipients may develop such a repertoire later or only after a delayed booster immunization, however, the current studies do not allow us to assess such an effect for the tested formulations. Convalescent sera have been used previously with varying success to treat LF infections (69–71) although passive protection studies using human or animal immune sera have been shown to protect guinea pigs and NHP from lethal infection (72–75). Furthermore, immunotherapy with a single or a cocktail of cross-neutralizing mAbs has also successfully protected and rescued guinea pigs and NHP from LASV infection (48, 76, 77).

It is uncertain if soluble GP can generate neutralizing antibodies with as few as two doses. A study using New Zealand White rabbits immunized with VLPs displaying LASV GP showed that high levels of LASV GP-surrogate neutralizing antibodies were induced only when 4 doses were administered (78). This could be an effect of the number of doses, dosing intervals or B-cell maturation from initial immunization. Macaques immunized with two doses of rVSV-ΔG-LASV-GP generated both protective cellular and humoral immune responses, including neutralizing antibodies, which were associated with survival against challenge (79), while two separate DNA vaccine studies also demonstrated LASV GP-specific neutralizing antibodies after two doses in surviving Guinea pigs and NHP (16, 17). Both vaccine platforms express the GP in its native form. Structural modifications to preserve the pre-fusion conformation have been described by Hastie et al. and include two key modifications: an intramolecular disulfide bond linking the GP1 and GP2 together and a helix-breaking proline in heptad region 1 (80). Trimerization domains, e.g., a foldon or isoleucine zipper, have also been used to promote protomer formation and generate a more homogenous oligomerization in recombinant glycoproteins of other viruses (81–84). Mechanistic modifications to enhance or maintain higher order protein structures may enhance the immune response to a recombinant subunit antigen and may be a prerequisite for reliable induction of neutralizing antibodies.

Historically, a robust CD4^+^ and CD8^+^ T-cell response during early infection has been associated with recovery from LF. This may develop into long-term polyfunctional T-cell memory (85–87). CD4^+^ T cell clones generated from lymphocytes from seven LASV seropositive individuals showed IFN-γ secretion upon stimulation with recombinant GP2, and CD8^+^ T cells from PBMCs collected from six LF survivors were activated by stimulation with GP antigen (27). These results indicate that the GP harbors important T-cell epitopes that correlate with survival. Similarly, several preclinical LF vaccines, e.g., vaccinia Ankara-LASV, MOPV-LASV, and the rVSV-ΔG-LASV-GP, have demonstrated activation, proliferation and cytokine secretion in response to LASV antigens by T-cells from protected mice, guinea pigs, and NHP with or without antibody production (3, 17, 53, 88–92).

We have shown that a two-dose regimen of recombinant LASV GP formulated with the adjuvants ISA 720, LiteVax or GPI-0100 induces a Th1-like cellular immunity which may be considered a correlate of protection against LASV infection, as well as IL-5 which is associated with decreased lethality seen in macaques surviving LASV infection from the Mali-strain (1, 54, 93). IFN-γ, TNF-α, IL-2, IL-4 and IL-5 were detected from murine splenocytes stimulated with GP antigens and/or peptides. The greater degree of IgG subclass diversity and cell-mediated cytokine secretion seen with LiteVax and GPI-0100 may be related to their intrinsic ability to activate T-cells *via* dendritic cells rather than solely antigen-mediated induction. Generally, 10 μg of antigen induced stronger responses than 2 μg, with 10 μg GP + GPI-0100 inducing the strongest overall T-cell response. We suspect that the greater abundance of antigen and the GPI-0100’s function as a surfactant may enable higher frequencies of lysosomal processing and presentation of the GP by APCs to lymphocytes, as well as promote NLRP3 activation which induces the release of Th1 cytokines (94). The type of response generated was similar to observations seen when this adjuvant was administered with the Herpes simplex virus type-1 (HSV-1) glycoprotein D (gD), HPV16 L2E6E7 fusion protein, Influenza A/PR8 (H1N1) subunit antigen, and the West Nile virus envelope protein (WN80E) in terms of antibody composition and cytokine secretion (40, 43, 95, 96). Animals immunized with GPI-0100 and a viral antigen developed greater numbers of IFN-γ, TNF-α, IL-4 and IL-5 secreting cells and were fully protected from viral challenge (95, 96), or showed improved clinical outcome (40, 43). While antigen dose-sparing was observed in terms of antibody titer and IgG subclass composition, especially with the LiteVax formulations, this was not the case with cytokine secretion as only mice receiving the higher antigen dose developed a polyfunctional cellular response, particularly with the GPI-0100 adjuvant.

Our results have demonstrated that a properly adjuvanted soluble LASV GP produced a robust humoral and cellular immune response in SW mice. ISA 51, ISA 720, LiteVax and GPI-0100 adjuvants in combination with the GP elicited potent antibody responses targeting the GP, with IgG1, IgG2a, and IgG2b subclasses in balance. These adjuvants have acceptable safety profiles and have been well-tolerated in clinical trials, with mostly localized and mild systemic adverse effects reported (30, 41, 97–102). This is encouraging as both saponin extracts and CFASE have been lyophilized without compromising immunostimulatory function (57, 103). This may enable the development of a thermostabilized vaccine which may be beneficial if a vaccine were to be deployed to areas where maintaining a cold chain is difficult as is the case for most LASV endemic areas. In summary, LASV protein subunit vaccines formulated with ISA 720, LiteVax, and GPI-0100 are promising candidates, with GPI-0100, in particular, generating a strong T-cell response in addition to potent humoral immunity.

## Supplementary Material

SUPPLEMENTARY MATERIAL

## Data Availability

The original contributions presented in the study are included in the article/Supplementary Material. Further inquiries can be directed to the corresponding author.
