# Supplementary material for: Adjuvants Differentially Modulate the Immunogenicity of Lassa Virus Glycoprotein Subunits in Mice
Source: Front Trop Dis. Author manuscript; Available in PMC 2023 Apr 7. (PMC10081732; doi:10.3389/fitd.2022.847598)

Supplementary Figure 1. (A) Representative Size-Exclusion Chromatography (SEC) Chromatogram and (B) Coomassie stained SDS-PAGE of SEC Fractions. The peak fractions from IAC purification with anti-LASV GP1 mAb R3621 were pooled and further purified using a HiLoad 16/600 SEC column equilibrated with PBS and separated at a linear flowrate of 1.0 mL/min. The elution was monitored by UV light absorption at 280 nm and fractions collected. The starting material (SM) and fractions 5-12 (F5-12) were mixed with sample loading dye for SDS-PAGE. Contents are as follows, F5-6: GPC protomer and oligomers, F7: GP2, trimer, F8: GP2 trimer and monomeric GP1, F9-12: monomeric GP1. Denaturing conditions resulted in the GP2 trimer dissociating into monomeric units resulting in lower band size in F7 and F8 in contrast to separation pattern on SEC. Fractions 5 and 6 were pooled for the final purified product.

SF1A

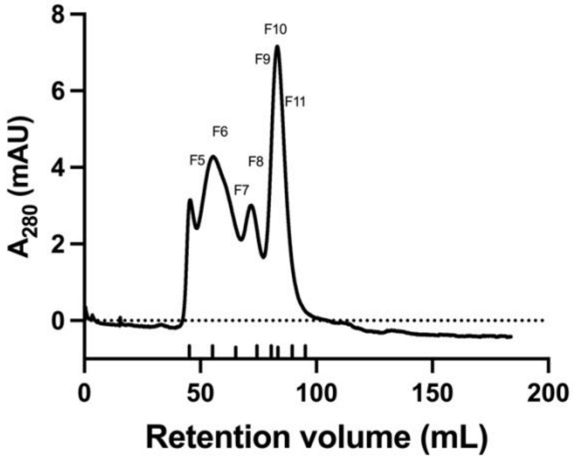

SF1B

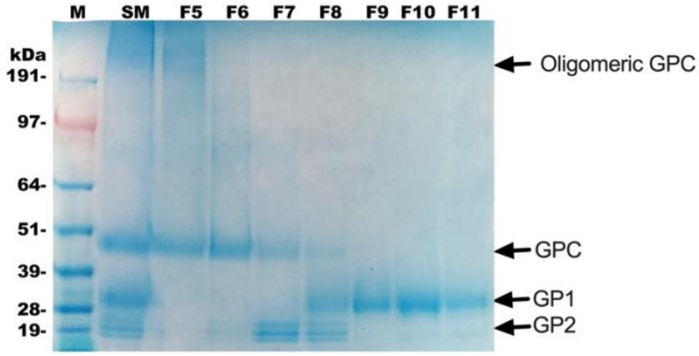

Supplementary Figure 2. Neutralizing Antibody Response to rVSV-LASV GP (A) Surrogate LASV neutralizing titers in pooled sera from mice immunized with adjuvanted LASV GP formulations collected 2 weeks after the 3rd dose were determined using a rVSV-LASV GP plaque reduction neutralization assay. (B) 19.7E, a known neutralizing mAb was used as a positive control starting at a concentration of 20 ug/mL. The data were graphed as a sigmoidal dose response using Graphpad Prism 9.

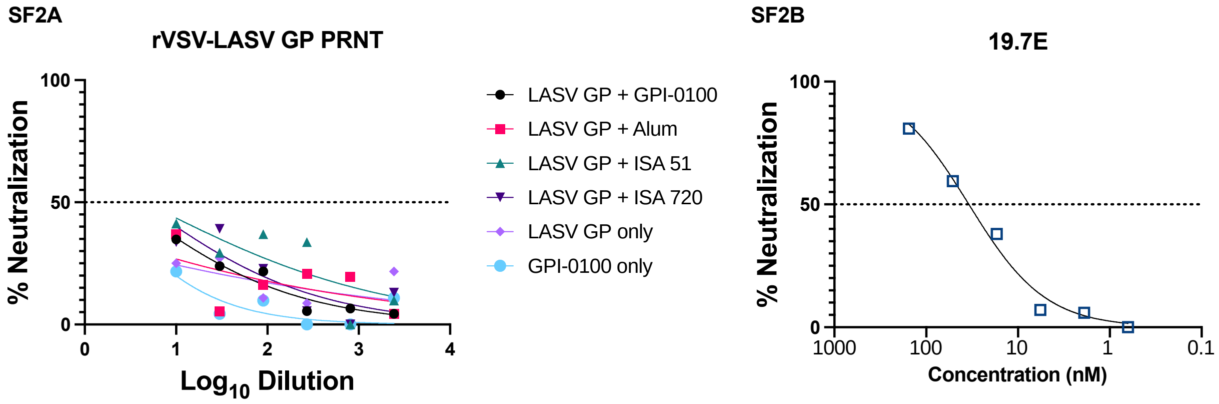

Supplement: SUPPLEMENTARY MATERIAL [file NIHMS1843995-supplement-SUPPLEMENTARY_MATERIAL.pdf]
